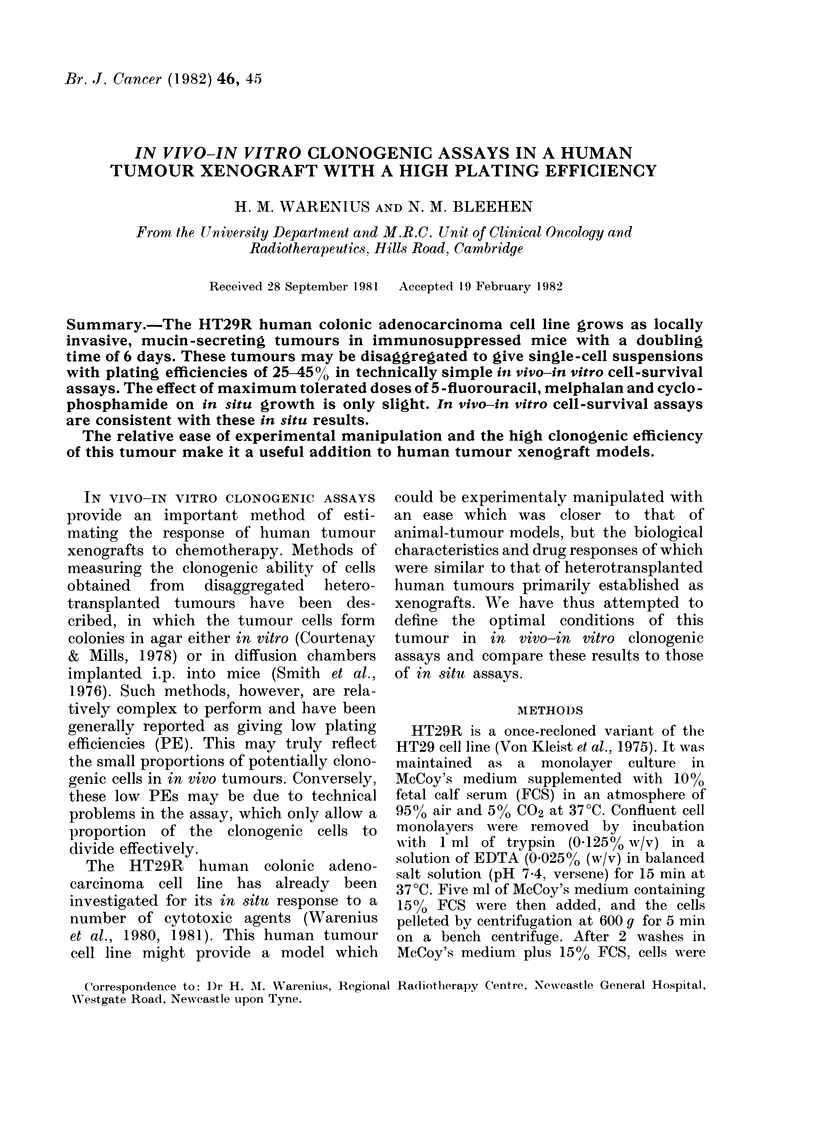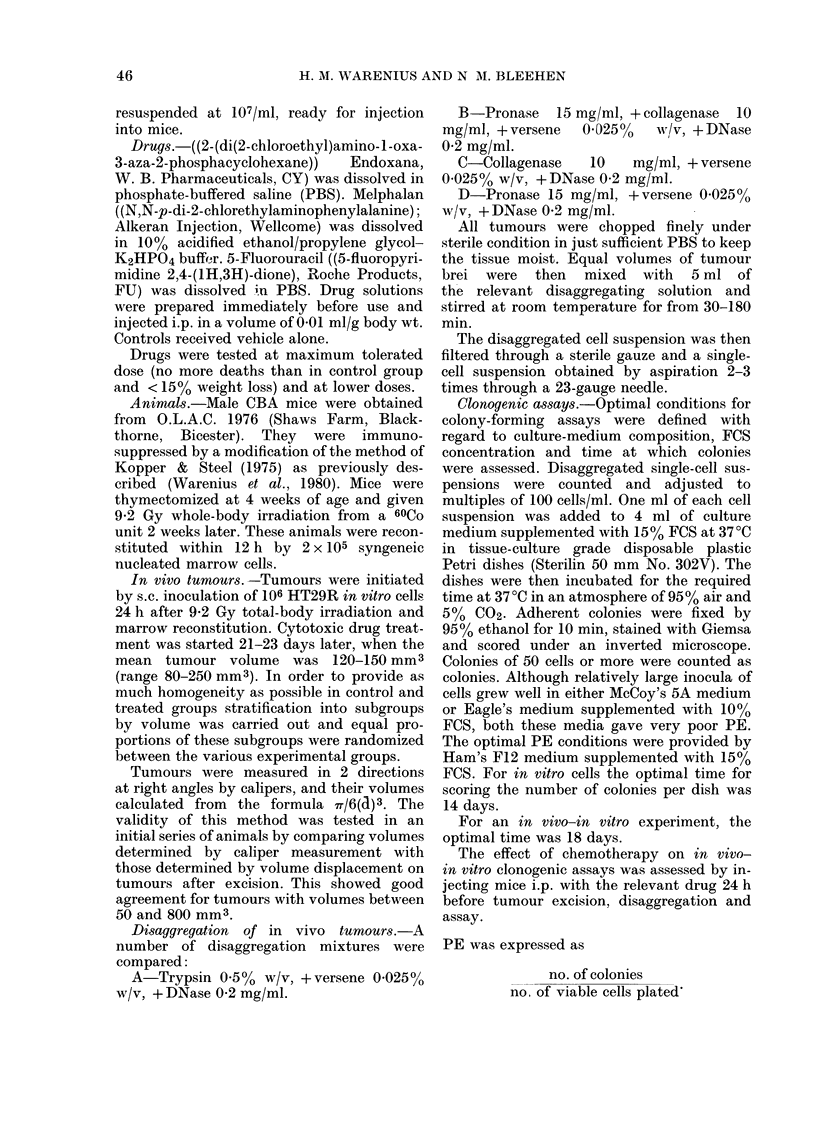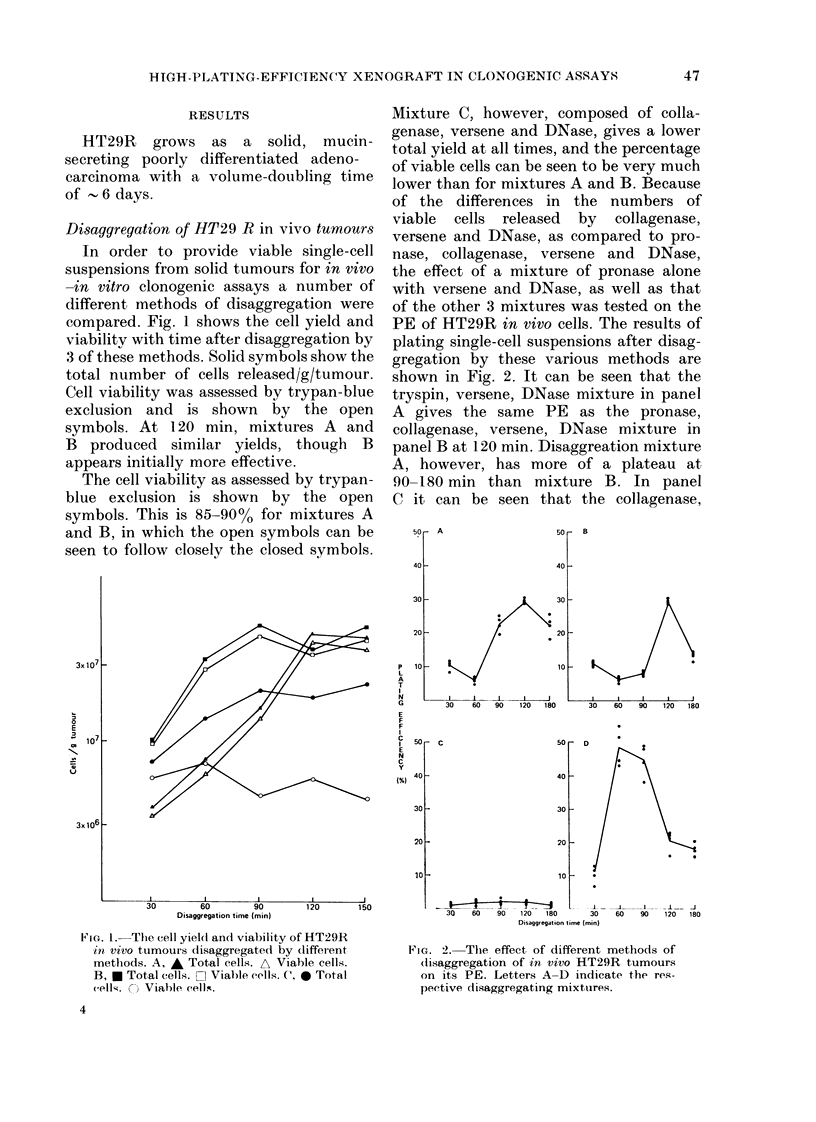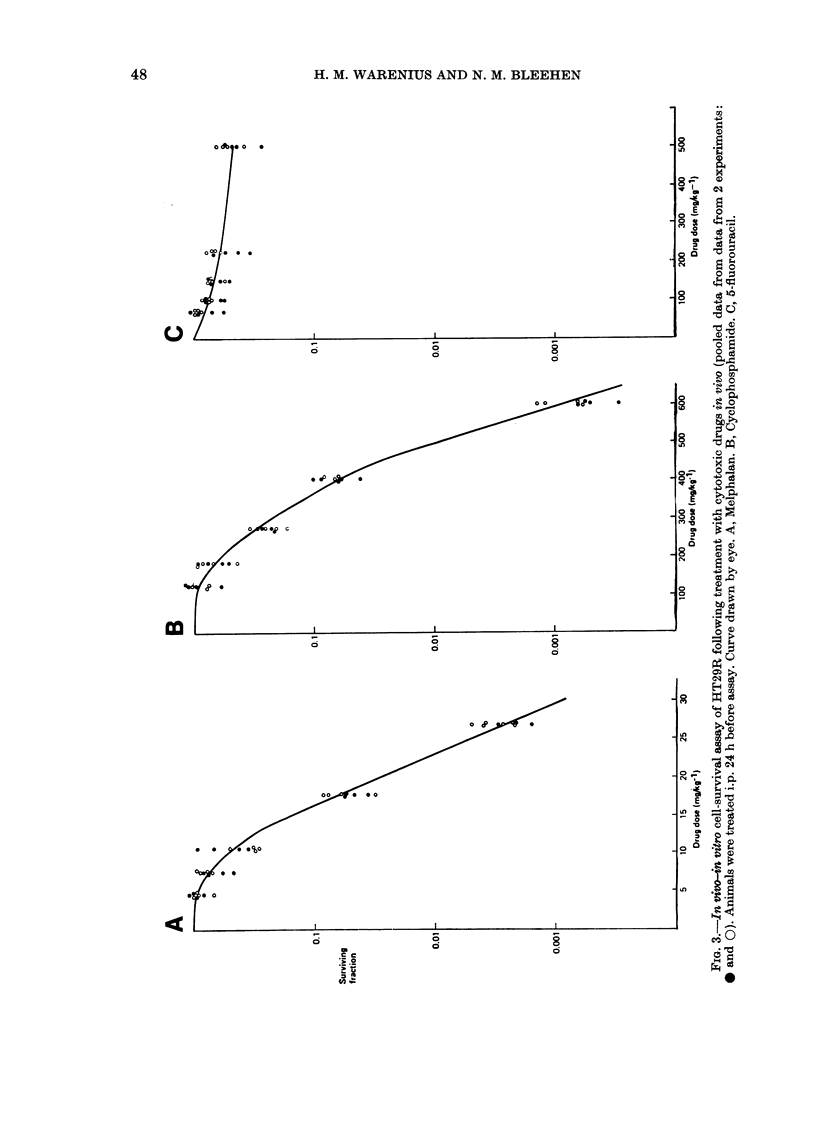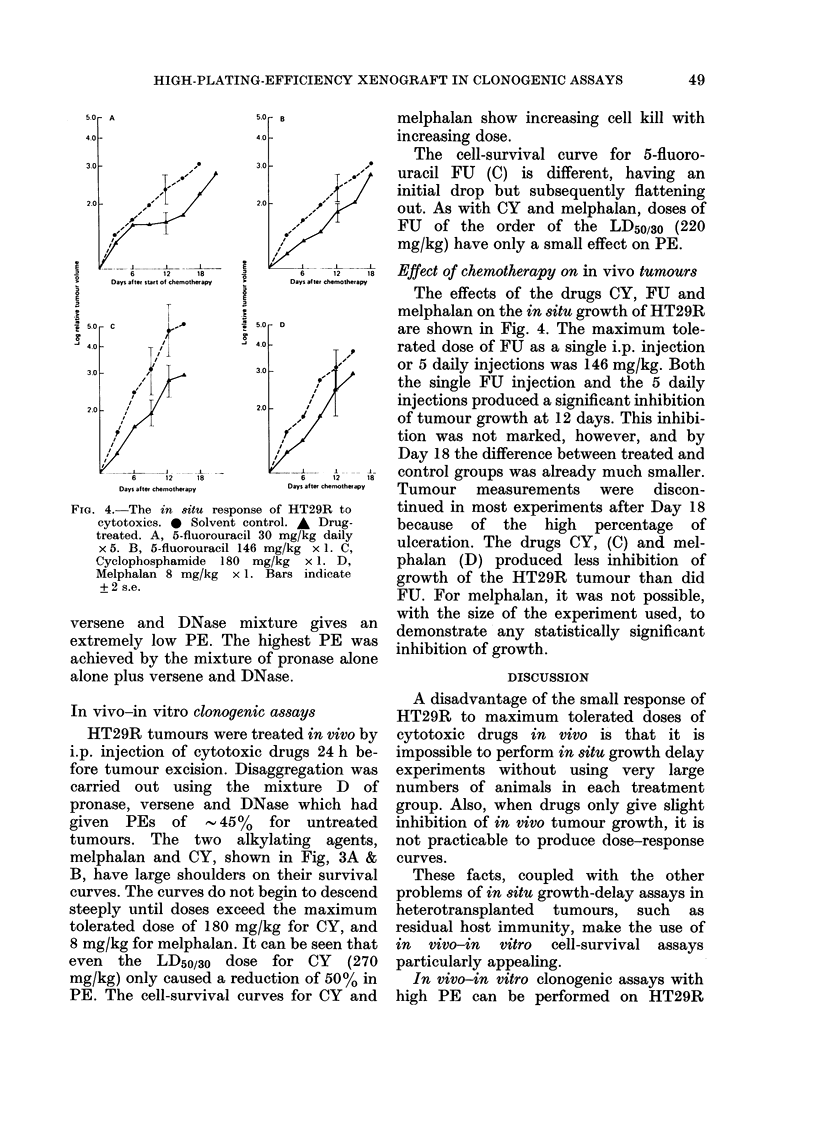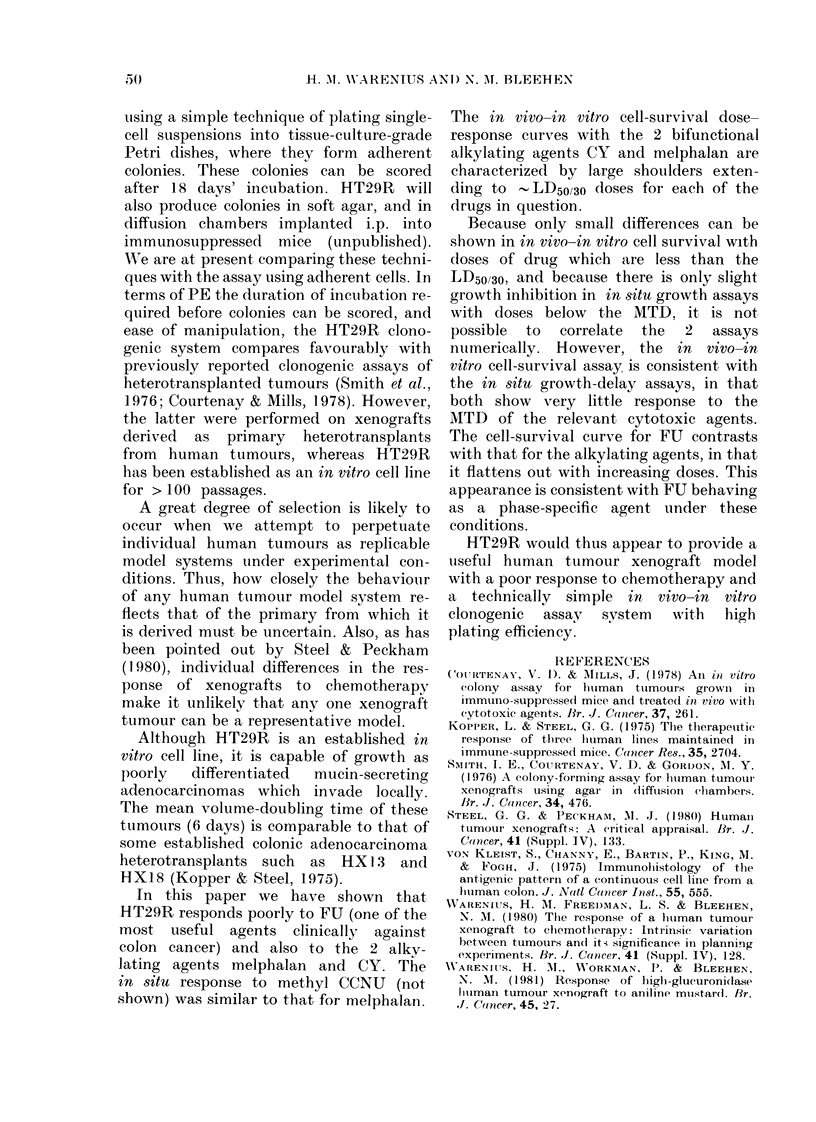# In vivo-in vitro clonogenic assays in a human tumour xenograft with a high plating efficiency.

**DOI:** 10.1038/bjc.1982.163

**Published:** 1982-07

**Authors:** H. M. Warenius, N. M. Bleehen

## Abstract

The HT29R human colonic adenocarcinoma cell line grows as locally invasive, mucin-secreting tumours in immunosuppressed mice with a doubling time of 6 days. These tumours may be disaggregated to give single-cell suspensions with plating efficiencies of 25-45% in technically simple in vivo-in vitro cell-survival assays. The effect of maximum tolerated doses of 5-fluorouracil, melphalan and cyclo-phosphamide on in situ growth is only slight. In vivo-in vitro cell-survival assays phosphamide on in situ growth is only slight. In vivo-in vitro cell-survival assays are consistent with these in situ results. The relative ease of experimental manipulation and the high clonogenic efficiency of this tumour make it a useful addition to human tumour xenograft models.


*Br. J. Cancer* (1982) **46**, 45

### *IN VIVO–IN VITRO* CLONOGENIC ASSAYS IN A HUMAN TUMOUR XENOGRAFT WITH A HIGH PLATING EFFICIENCY

H. M. WARENIUS AND N. M. BLEEHEN

*From the University Department and M.R.C. Unit of Clinical Oncology and Radiotherapeutics, Hills Road, Cambridge*

Received 28 September 1981 Accepted 19 February 1982

**Summary.—The HT29R human colonic adenocarcinoma cell line grows as locally invasive, mucin-secreting tumours in immunosuppressed mice with a doubling time of 6 days. These tumours may be disaggregated to give single-cell suspensions with plating efficiencies of 25–45% in technically simple *in vivo–in vitro* cell-survival assays. The effect of maximum tolerated doses of 5-fluorouracil, melphalan and cyclophosphamide on *in situ* growth is only slight. *In vivo–in vitro* cell-survival assays are consistent with these *in situ* results.**

**The relative ease of experimental manipulation and the high clonogenic efficiency of this tumour make it a useful addition to human tumour xenograft models.**

IN VIVO–IN VITRO CLONOGENIC ASSAYS provide an important method of estimating the response of human tumour xenografts to chemotherapy. Methods of measuring the clonogenic ability of cells obtained from disaggregated heterotransplanted tumours have been described, in which the tumour cells form colonies in agar either *in vitro* (Courtenay & Mills, 1978) or in diffusion chambers implanted i.p. into mice (Smith *et al.*, 1976). Such methods, however, are relatively complex to perform and have been generally reported as giving low plating efficiencies (PE). This may truly reflect the small proportions of potentially clonogenic cells in *in vivo* tumours. Conversely, these low PEs may be due to technical problems in the assay, which only allow a proportion of the clonogenic cells to divide effectively.

The HT29R human colonic adenocarcinoma cell line has already been investigated for its *in situ* response to a number of cytotoxic agents (Warenius *et al.*, 1980, 1981). This human tumour cell line might provide a model which could be experimentaly manipulated with an ease which was closer to that of animal-tumour models, but the biological characteristics and drug responses of which were similar to that of heterotransplanted human tumours primarily established as xenografts. We have thus attempted to define the optimal conditions of this tumour in *in vivo–in vitro* clonogenic assays and compare these results to those of *in situ* assays.

### METHODS

HT29R is a once-recloned variant of the HT29 cell line (Von Kleist *et al.*, 1975). It was maintained as a monolayer culture in McCoy's medium supplemented with 10% fetal calf serum (FCS) in an atmosphere of 95% air and 5% $CO_2$ at 37 °C. Confluent cell monolayers were removed by incubation with 1 ml of trypsin (0·125% w/v) in a solution of EDTA (0·025% (w/v) in balanced salt solution (pH 7·4, versene) for 15 min at 37 °C. Five ml of McCoy's medium containing 15% FCS were then added, and the cells pelleted by centrifugation at 600 *g* for 5 min on a bench centrifuge. After 2 washes in McCoy's medium plus 15% FCS, cells were

Correspondence to: Dr H. M. Warenius, Regional Radiotherapy Centre, Newcastle General Hospital, Westgate Road, Newcastle upon Tyne.

resuspended at $10^7$/ml, ready for injection into mice.

*Drugs.*—((2-(di(2-chloroethyl)amino-1-oxa-3-aza-2-phosphacyclohexane)) Endoxana, W. B. Pharmaceuticals, CY) was dissolved in phosphate-buffered saline (PBS). Melphalan ((N,N-*p*-di-2-chlorethylaminophenylalanine); Alkeran Injection, Wellcome) was dissolved in 10% acidified ethanol/propylene glycol–$K_2HPO_4$ buffer. 5-Fluorouracil ((5-fluoropyrimidine 2,4-(1H,3H)-dione), Roche Products, FU) was dissolved in PBS. Drug solutions were prepared immediately before use and injected i.p. in a volume of 0·01 ml/g body wt. Controls received vehicle alone.

Drugs were tested at maximum tolerated dose (no more deaths than in control group and $<15\%$ weight loss) and at lower doses.

*Animals.*—Male CBA mice were obtained from O.L.A.C. 1976 (Shaws Farm, Blackthorne, Bicester). They were immunosuppressed by a modification of the method of Kopper & Steel (1975) as previously described (Warenius *et al.*, 1980). Mice were thymectomized at 4 weeks of age and given 9·2 Gy whole-body irradiation from a $^{60}$Co unit 2 weeks later. These animals were reconstituted within 12 h by $2 \times 10^5$ syngeneic nucleated marrow cells.

*In vivo tumours.*—Tumours were initiated by s.c. inoculation of $10^6$ HT29R *in vitro* cells 24 h after 9·2 Gy total-body irradiation and marrow reconstitution. Cytotoxic drug treatment was started 21–23 days later, when the mean tumour volume was 120–150 mm$^3$ (range 80–250 mm$^3$). In order to provide as much homogeneity as possible in control and treated groups stratification into subgroups by volume was carried out and equal proportions of these subgroups were randomized between the various experimental groups.

Tumours were measured in 2 directions at right angles by calipers, and their volumes calculated from the formula $\pi/6(\bar{d})^3$. The validity of this method was tested in an initial series of animals by comparing volumes determined by caliper measurement with those determined by volume displacement on tumours after excision. This showed good agreement for tumours with volumes between 50 and 800 mm$^3$.

*Disaggregation of* in vivo *tumours.*—A number of disaggregation mixtures were compared:

A—Trypsin 0·5% w/v, +versene 0·025% w/v, +DNase 0·2 mg/ml.

B—Pronase 15 mg/ml, +collagenase 10 mg/ml, +versene 0·025% w/v, +DNase 0·2 mg/ml.

C—Collagenase 10 mg/ml, +versene 0·025% w/v, +DNase 0·2 mg/ml.

D—Pronase 15 mg/ml, +versene 0·025% w/v, +DNase 0·2 mg/ml.

All tumours were chopped finely under sterile condition in just sufficient PBS to keep the tissue moist. Equal volumes of tumour brei were then mixed with 5 ml of the relevant disaggregating solution and stirred at room temperature for from 30–180 min.

The disaggregated cell suspension was then filtered through a sterile gauze and a single-cell suspension obtained by aspiration 2–3 times through a 23-gauge needle.

*Clonogenic assays.*—Optimal conditions for colony-forming assays were defined with regard to culture-medium composition, FCS concentration and time at which colonies were assessed. Disaggregated single-cell suspensions were counted and adjusted to multiples of 100 cells/ml. One ml of each cell suspension was added to 4 ml of culture medium supplemented with 15% FCS at 37 °C in tissue-culture grade disposable plastic Petri dishes (Sterilin 50 mm No. 302V). The dishes were then incubated for the required time at 37 °C in an atmosphere of 95% air and 5% $CO_2$. Adherent colonies were fixed by 95% ethanol for 10 min, stained with Giemsa and scored under an inverted microscope. Colonies of 50 cells or more were counted as colonies. Although relatively large inocula of cells grew well in either McCoy's 5A medium or Eagle's medium supplemented with 10% FCS, both these media gave very poor PE. The optimal PE conditions were provided by Ham's F12 medium supplemented with 15% FCS. For *in vitro* cells the optimal time for scoring the number of colonies per dish was 14 days.

For an *in vivo–in vitro* experiment, the optimal time was 18 days.

The effect of chemotherapy on *in vivo–in vitro* clonogenic assays was assessed by injecting mice i.p. with the relevant drug 24 h before tumour excision, disaggregation and assay.

PE was expressed as

$$\frac{\text{no. of colonies}}{\text{no. of viable cells plated}}.$$

### RESULTS

HT29R grows as a solid, mucin-secreting poorly differentiated adenocarcinoma with a volume-doubling time of ~6 days.

### *Disaggregation of HT29 R* in vivo *tumours*

In order to provide viable single-cell suspensions from solid tumours for *in vivo -in vitro* clonogenic assays a number of different methods of disaggregation were compared. Fig. 1 shows the cell yield and viability with time after disaggregation by 3 of these methods. Solid symbols show the total number of cells released/g/tumour. Cell viability was assessed by trypan-blue exclusion and is shown by the open symbols. At 120 min, mixtures A and B produced similar yields, though B appears initially more effective.

The cell viability as assessed by trypan-blue exclusion is shown by the open symbols. This is 85–90% for mixtures A and B, in which the open symbols can be seen to follow closely the closed symbols. Mixture C, however, composed of collagenase, versene and DNase, gives a lower total yield at all times, and the percentage of viable cells can be seen to be very much lower than for mixtures A and B. Because of the differences in the numbers of viable cells released by collagenase, versene and DNase, as compared to pronase, collagenase, versene and DNase, the effect of a mixture of pronase alone with versene and DNase, as well as that of the other 3 mixtures was tested on the PE of HT29R *in vivo* cells. The results of plating single-cell suspensions after disaggregation by these various methods are shown in Fig. 2. It can be seen that the tryspin, versene, DNase mixture in panel A gives the same PE as the pronase, collagenase, versene, DNase mixture in panel B at 120 min. Disaggreation mixture A, however, has more of a plateau at 90–180 min than mixture B. In panel C it can be seen that the collagenase,

FIG. 1.—The cell yield and viability of HT29R *in vivo* tumours disaggregated by different methods. A, ▲ Total cells. △ Viable cells. B, ■ Total cells. □ Viable cells. C, ● Total cells. ○ Viable cells.

FIG. 2.—The effect of different methods of disaggregation of *in vivo* HT29R tumours on its PE. Letters A–D indicate the respective disaggregating mixtures.

FIG. 3.—*In vivo–in vitro* cell-survival assay of HT29R following treatment with cytotoxic drugs *in vivo* (pooled data from data from 2 experiments: ● and ○). Animals were treated i.p. 24 h before assay. Curve drawn by eye. A, Melphalan. B, Cyclophosphamide. C, 5-fluorouracil.

FIG. 4.—The *in situ* response of HT29R to cytotoxics. ● Solvent control. ▲ Drug-treated. A, 5-fluorouracil 30 mg/kg daily ×5. B, 5-fluorouracil 146 mg/kg ×1. C, Cyclophosphamide 180 mg/kg ×1. D, Melphalan 8 mg/kg ×1. Bars indicate ±2 s.e.

versene and DNase mixture gives an extremely low PE. The highest PE was achieved by the mixture of pronase alone alone plus versene and DNase.

### In vivo–in vitro *clonogenic assays*

HT29R tumours were treated *in vivo* by i.p. injection of cytotoxic drugs 24 h before tumour excision. Disaggregation was carried out using the mixture D of pronase, versene and DNase which had given PEs of ~45% for untreated tumours. The two alkylating agents, melphalan and CY, shown in Fig, 3A & B, have large shoulders on their survival curves. The curves do not begin to descend steeply until doses exceed the maximum tolerated dose of 180 mg/kg for CY, and 8 mg/kg for melphalan. It can be seen that even the $LD_{50/30}$ dose for CY (270 mg/kg) only caused a reduction of 50% in PE. The cell-survival curves for CY and melphalan show increasing cell kill with increasing dose.

The cell-survival curve for 5-fluorouracil FU (C) is different, having an initial drop but subsequently flattening out. As with CY and melphalan, doses of FU of the order of the $LD_{50/30}$ (220 mg/kg) have only a small effect on PE.

### *Effect of chemotherapy on* in vivo *tumours*

The effects of the drugs CY, FU and melphalan on the *in situ* growth of HT29R are shown in Fig. 4. The maximum tolerated dose of FU as a single i.p. injection or 5 daily injections was 146 mg/kg. Both the single FU injection and the 5 daily injections produced a significant inhibition of tumour growth at 12 days. This inhibition was not marked, however, and by Day 18 the difference between treated and control groups was already much smaller. Tumour measurements were discontinued in most experiments after Day 18 because of the high percentage of ulceration. The drugs CY, (C) and melphalan (D) produced less inhibition of growth of the HT29R tumour than did FU. For melphalan, it was not possible, with the size of the experiment used, to demonstrate any statistically significant inhibition of growth.

### DISCUSSION

A disadvantage of the small response of HT29R to maximum tolerated doses of cytotoxic drugs *in vivo* is that it is impossible to perform *in situ* growth delay experiments without using very large numbers of animals in each treatment group. Also, when drugs only give slight inhibition of *in vivo* tumour growth, it is not practicable to produce dose–response curves.

These facts, coupled with the other problems of *in situ* growth-delay assays in heterotransplanted tumours, such as residual host immunity, make the use of *in vivo–in vitro* cell-survival assays particularly appealing.

*In vivo–in vitro* clonogenic assays with high PE can be performed on HT29R

using a simple technique of plating single-cell suspensions into tissue-culture-grade Petri dishes, where they form adherent colonies. These colonies can be scored after 18 days' incubation. HT29R will also produce colonies in soft agar, and in diffusion chambers implanted i.p. into immunosuppressed mice (unpublished). We are at present comparing these techniques with the assay using adherent cells. In terms of PE the duration of incubation required before colonies can be scored, and ease of manipulation, the HT29R clonogenic system compares favourably with previously reported clonogenic assays of heterotransplanted tumours (Smith *et al.*, 1976; Courtenay & Mills, 1978). However, the latter were performed on xenografts derived as primary heterotransplants from human tumours, whereas HT29R has been established as an *in vitro* cell line for $>100$ passages.

A great degree of selection is likely to occur when we attempt to perpetuate individual human tumours as replicable model systems under experimental conditions. Thus, how closely the behaviour of any human tumour model system reflects that of the primary from which it is derived must be uncertain. Also, as has been pointed out by Steel & Peckham (1980), individual differences in the response of xenografts to chemotherapy make it unlikely that any one xenograft tumour can be a representative model.

Although HT29R is an established *in vitro* cell line, it is capable of growth as poorly differentiated mucin-secreting adenocarcinomas which invade locally. The mean volume-doubling time of these tumours (6 days) is comparable to that of some established colonic adenocarcinoma heterotransplants such as HX13 and HX18 (Kopper & Steel, 1975).

In this paper we have shown that HT29R responds poorly to FU (one of the most useful agents clinically against colon cancer) and also to the 2 alkylating agents melphalan and CY. The *in situ* response to methyl CCNU (not shown) was similar to that for melphalan. The *in vivo–in vitro* cell-survival dose–response curves with the 2 bifunctional alkylating agents CY and melphalan are characterized by large shoulders extending to $\sim LD_{50/30}$ doses for each of the drugs in question.

Because only small differences can be shown in *in vivo–in vitro* cell survival with doses of drug which are less than the $LD_{50/30}$, and because there is only slight growth inhibition in *in situ* growth assays with doses below the MTD, it is not possible to correlate the 2 assays numerically. However, the *in vivo–in vitro* cell-survival assay is consistent with the *in situ* growth-delay assays, in that both show very little response to the MTD of the relevant cytotoxic agents. The cell-survival curve for FU contrasts with that for the alkylating agents, in that it flattens out with increasing doses. This appearance is consistent with FU behaving as a phase-specific agent under these conditions.

HT29R would thus appear to provide a useful human tumour xenograft model with a poor response to chemotherapy and a technically simple *in vivo–in vitro* clonogenic assay system with high plating efficiency.

### REFERENCES

Courtenay, V. D. & Mills, J. (1978) An *in vitro* colony assay for human tumours grown in immuno-suppressed mice and treated *in vivo* with cytotoxic agents. *Br. J. Cancer*, **37**, 261.

Kopper, L. & Steel, G. G. (1975) The therapeutic response of three human lines maintained in immune-suppressed mice. *Cancer Res.*, **35**, 2704.

Smith, I. E., Courtenay, V. D. & Gordon, M. Y. (1976) A colony-forming assay for human tumour xenografts using agar in diffusion chambers. *Br. J. Cancer*, **34**, 476.

Steel, G. G. & Peckham, M. J. (1980) Human tumour xenografts: A critical appraisal. *Br. J. Cancer*, **41** (Suppl. IV), 133.

von Kleist, S., Channy, E., Bartin, P., King, M. & Fogh, J. (1975) Immunohistology of the antigenic pattern of a continuous cell line from a human colon. *J. Natl Cancer Inst.*, **55**, 555.

Warenius, H. M. Freedman, L. S. & Bleehen, N. M. (1980) The response of a human tumour xenograft to chemotherapy: Intrinsic variation between tumours and its significance in planning experiments. *Br. J. Cancer*, **41** (Suppl. IV), 128.

Warenius, H. M., Workman, P. & Bleehen, N. M. (1981) Response of high-glucuronidase human tumour xenograft to aniline mustard. *Br. J. Cancer*, **45**, 27.